# Evaluation of the accuracy of the CellaVision™ DM96 in a high HIV-prevalence population in South Africa

**DOI:** 10.4102/ajlm.v5i1.313

**Published:** 2016-03-09

**Authors:** Jenifer L. Vaughan, Sakina Loonat, Nazeer Alli

**Affiliations:** 1 Department of Molecular Medicine and Haematology, University of the Witwatersrand, Johannesburg, South Africa; 2 Department of Haematology at the Chris Hani Baragwanath Academic Hospital, National Health Laboratory Services, Johannesburg, South Africa

## Abstract

**Introduction:**

The CellaVision™ DM96 (DM96) is a digital microscopy system which performs well in developed countries. However, to date it has not been evaluated in Africa, where the pathology spectrum encountered is very different. In particular, its utility in a setting with high HIV prevalence has not been assessed, which is of interest because of the morphological aberrations often seen in HIV-positive patients.

**Objectives:**

This study aimed to evaluate the accuracy of the DM96 in a South African laboratory, with emphasis on its performance in samples collected from HIV-positive patients.

**Methods:**

A total of 149 samples submitted for a routine differential white cell count in 2012 and 2013 at the Chris Hani Baragwanath Academic Hospital in Johannesburg, South Africa were included, of which 79 (53.0%) were collected from HIV-positive patients. Results of DM96 analysis pre- and post-classification were compared with a manual differential white cell count and the impact of HIV infection and other variables of interest were assessed.

**Results:**

Pre- and post-classification accuracies were similar to those reported in developed countries. Reclassification was required in 16% of cells, with particularly high misclassification rates for eosinophils (31.7%), blasts (33.7%) and basophils (93.5%). Multivariate analysis revealed a significant relationship between the number of misclassified cells and both the white cell count (*p* = 0.035) and the presence of malignant cells in the blood (*p* = 0.049), but not with any other variables analysed, including HIV status.

**Conclusion:**

The DM96 exhibited acceptable accuracy in this South African laboratory, which was not impacted by HIV infection. However, as it does not eliminate the need for experienced morphologists, its cost may be unjustifiable in a resource-constrained setting.

## Introduction

The differential white cell count (DWCC) is a frequently requested laboratory investigation and is, historically, a labour-intensive test. Although the guidelines established by the international consensus group for haematology to identify samples where peripheral smear review may be omitted^1^ have considerably reduced the workload on the morphology bench, manual smear review remains necessary in a substantial proportion of cases. With the number of skilled medical technologists on the decline worldwide, morphology skills are becoming ever scarcer, particularly in Africa where laboratory resources are, in general, grossly strained.^2,3^ Novis et al. reported the smear review rate to be proportional to the number of occupied beds in the hospital served,^4^ which they speculated to reflect a higher pathology burden in larger hospitals. In sub-Saharan Africa, the pathology burden (and hence the need for smear review) is compounded by the HIV epidemic, which places an extra strain on haematology services because of the many haematological complications of HIV infection. There is thus a dire need for analysers that can improve laboratory efficiency in this setting.

The CellaVision™ DM96 (CellaVision AB, Lund, Sweden; hereafter, ‘DM96’) is a digital microscopy system that has the potential to minimise the time required by a morphologist to perform a manual DWCC. It comprises: an automated microscope that scans the blood smear; a digital camera that captures images of all the cellular and particulate material on the slide; and a computer that classifies each image by means of complex algorithms. It has been demonstrated to have good performance characteristics in developed countries,^5,6,7,8,9^ but to date has not been evaluated in Africa, where the spectrum of pathology encountered is very different. Most notable in sub-Saharan Africa is the extremely high prevalence of HIV infection. More than 25.5 million people live with HIV in this region, compared with less than 2.5 million in Western/Central Europe and North America combined.^10^ The effect of this epidemic is greatest in communities with a poor socio-economic background. In South Africa, the most substantial impact is on state-sector hospitals. Because HIV infection is often associated with a number of morphological peculiarities of the white cells, including the frequent presence of atypical activated lymphocytes and abnormal nucleation of the neutrophils,^11^ its impact on the performance of the DM96 is of interest for laboratories operating in areas with high HIV prevalence. The Chris Hani Baragwanath Academic Hospital (CHBAH) is a large referral centre in Johannesburg, South Africa, which serves a community with a poor socio-economic background and high HIV prevalence. The aim of this study was to assess the accuracy of the DWCC generated by the DM96 in the CHBAH laboratory, with emphasis on its performance in samples collected from HIV-positive patients.

## Research method and design

### Ethical considerations

Ethical clearance was obtained from the Human Research Ethics Committee of the University of the Witwatersrand, Johannesburg, South Africa (clearance number: M090688).

### Sample selection and analysis

The study was performed at the National Health Laboratory Service (NHLS) haematology laboratory at CHBAH in Soweto, Johannesburg, South Africa. A total of 149 peripheral blood samples were selected from EDTA-anticoagulated specimens submitted for a DWCC over the course of 2012 and 2013. Samples were selected to cover a wide range of white cell counts and were included only if the patient had an HIV test result available in the laboratory information system (LIS) (DisaLab Version 04.16.04.373, Laboratory System Technologies, Boksburg, Gauteng, South Africa). Slides were made and stained with May-Grünwald/Giemsa by an automated slide maker and stainer (SP-100, Sysmex, Kobe, Japan) as per standard operating procedure for the performance of a DWCC. Each smear was examined by an experienced morphologist and analysed with the DM96 within a 24-hour period. The manual DWCC was performed on 100 cells. The DM96 was set to analyse 110 images per sample. Each image was pre-classified as an unidentified cell, a neutrophil, a lymphocyte, a monocyte, a granulocyte precursor (i.e., a promyelocyte, myelocyte or metamyelocyte), a blast, an eosinophil, a basophil, a nucleated red cell, a giant platelet, a platelet aggregate, a smear cell or an artefact. Cells which the instrument identified as being band cells were classified as neutrophils. The images were then viewed by the same morphologist who performed the manual DWCC. The morphologist either verified the DM96 pre-classification or reclassified the cells. Hereafter, the initial DWCC performed by the DM96 will be referred to as the ‘pre-classification DWCC’ and the final DWCC following review by the morphologist will be referred to as the ‘post-classification DWCC’. ‘Misclassification’ will refer to cells requiring reclassification by the morphologist.

For each sample, the data available in the LIS were reviewed and pertinent information recorded. This included the clinical information provided by the attending clinician, demographic details, evidence of infection (including C-reactive protein levels and culture results), recent HIV viral load and CD4 counts (where appropriate), exposure to anti-retroviral therapy, as well as bone marrow aspirate/trephine biopsy and other histology findings. The data were recorded in Excel™ spreadsheets (Microsoft Office Excel™ 2007, Redmond, Washington, United States). No identifying patient information was recorded. All data collection and analysis were performed by a haematopathologist working in the haematology laboratory of the CHBAH.

### Statistical analysis

Demographic data are presented as medians (interquartile range [IQR]), mean (± standard deviation [SD]) and proportions, as appropriate. The accuracy of the DM96 DWCC was evaluated by linear regression (pre- and post-classification) and Bland-Altman (post-classification) analyses comparing the absolute count for neutrophils, monocytes, lymphocytes, eosinophils, basophils and blasts with those obtained by manual counting. The misclassification rate was determined as the proportion of all counted cells requiring reclassification. A multivariate linear regression analysis was performed to assess the impact of variables of interest, including HIV infection, on misclassification rates. Any data point with a standard residual of greater than 2.5 was excluded from analysis and *p*-values less than 0.05 were considered statistically significant. Statistical analysis was performed using STATISTICA software, version 12.5 (Stat Soft [Pty] Ltd; Tulsa, Oklahoma, United States).

## Results

Patient demographic and clinical data are summarised in Table 1. The median white cell count (WCC) was 6.76 × 10^9^/L (range 0.28–262). The WCC was < 1.5 × 10^9^/L in 14 patients (9.4%) and > 50 × 10^9^/L in 12 patients (8.1%). Slightly over half (*n* = 79; 53%) of patients were HIV-positive, whilst ~40% (*n* = 61) had a history of malignancy. The prevalence of malignancy was similar between HIV-positive and HIV-negative patients, although the spectrum of malignant disease varied substantially between these two groups. The dominant malignancy amongst HIV-positive patients was high-grade lymphomas, whereas leukaemias were more common amongst HIV-negative patients. Despite this difference, the proportion of patients with abnormal cells present in the peripheral blood was similar between the two groups. Not surprisingly, evidence of infection was substantially more common amongst HIV-positive patients.

**TABLE 1:** Patient demographic and clinical data for samples included in study, Chris Hani Baragwanath Academic Hospital haematology laboratory, Johannesburg, South Africa, 2012–2013 (*N* = 149).

Parameter	All patients (*N*=149)	HIV-positive (*n*=79)	HIV-negative (*n*=70)
Age (years) (median [IQR]) (*n* = 147)	40 (30–52)†	38 (31–45)	47 (22–58)‡
CD4 count (× 10^6^/L) (median [IQR]) (*n* = 52)	129 (39–331)	129 (39–331)	N/A
HIVVL (copies/mL) (median [IQR]) (*n* = 30)	150 (40–86 910)	150 (40–86 910)	N/A
ART (yes/no)	35/8	35/8	N/A
History of malignancy (*n* [%])	61 (41.6)	35 (44.3)	26 (36.6)
High-grade lymphoma (*n* [%])	23 (37.7)	22 (62.9)	1 (3.8)
Acute leukaemia (*n* [%])	12 (19.7)	2 (5.7)	10 (38.5)
Chronic myeloid leukaemia (*n* [%])	11 (18.0)	5 (14.3)	6 (23.1)
Low grade lympho-proliferative disorders (*n* [%])	5 (8.2)	1 (2.9)	4 (15.4)
Other (*n* [%])	10 (16.4)	5 (14.3)	5 (19.2)
Recent chemotherapy	43 (28.9)	20 (25.3)	23 (32.4)
Malignant cells in the blood (*n* [%])	17 (11.4)	8 (10.1)	9 (12.7)
Evidence of infection (*n* [%])	48 (32.2)	33 (41.8)	15 (21.1)

WCC, White cell count; IQR, Interquartile range; N/A, not applicable; HIVVL, HIV viral load; ART, anti-retroviral therapy.

† *n* = 147 (age was not provided for two patients);

‡ *n* = 69.

### Analysis of accuracy

Overall, pre-classification DWCC accuracy was very poor, with good correlation occurring only for neutrophils (Table 2). Correlation co-efficient (CC) values improved substantially post-classification, but remained below 0.9 for all cell types except neutrophils and blasts, and was poor for eosinophils, basophils and monocytes. However, the Bland-Altman analysis showed that agreement was within acceptable limits of bias for neutrophils, lymphocytes and blasts, with borderline acceptable agreement for monocytes. A substantial negative bias was evident for both eosinophils and basophils, but because of the low levels of these cell types, this translated into small differences in absolute values, the clinical significance of which was negligible.

**TABLE 2:** Accuracy of CellaVision™ DM96 analyser, Chris Hani Baragwanath Academic Hospital haematology laboratory, Johannesburg, South Africa, 2012–2013.

Leukocyte subset	Pre-classification DWCC accuracy† (*r*^2^ [slope/intercept])	Post-classification DWCC accuracy‡ (*r*^2^ [slope/intercept])	Bias (absolute (%) [Limits of agreement])	Allowable bias§ (%)	Absolute manual count (× 10^9^/L) (mean [±SD])	Absolute DM96 count (× 10^9^/L) (mean [±SD])
Neutrophils	0.99 (1.04/-0.61)	0.99 (1.01/-0.61)	0.58 (4.5) (-6.0–7.2)	9.25	12.3 (±23.4)	12.9 (±25.8)
Lymphocytes	0.25 (1.1/1.1)	0.86 (1.04/0.07)	0.15 (8.1) (-2.7–3.0)	9.19	1.81 (±3.4)	1.96 (±3.8)
Monocytes	0.35 (0.4/0.2)	0.51 (0.53/0.24)	-0.12 (-16.8) (-2.2–2.0)	13.2	0.78 (±1.5)	0.66 (±1.4)
Eosinophils	0.65 (0.7/0.06)	0.72 (0.58/0.48)	-0.11 (-35.7) (-1.59–1.36)	19.8	0.36 (±1.34)	0.25 (±0.92)
Basophils	0.26 (0.04/0.02)	0.64 (0.33/0.1)	-0.22 (-58.2) (-4–3.59)	15.4	0.48 (±2.7)	0.27 (±1.1)
Blasts	0.79 (0.3/0.1)	0.99 (0.97/0.06)	-0.08 (-3.7) (-2.9–2.74)	-	2.13 (±16.4)	2.05 (±15.3)

DWCC, Differential white cell count.

† Pre-classification DWCC refers to the DM96 analyser results before review by the morphologist who performed a manual DWCC;

‡ Post-classification DWCC refers to the DM96 analyser results after review and reclassification by the morphologist who performed a manual DWCC of the same sample. Results derived from linear regression, which compared the absolute counts for neutrophils, monocytes, lymphocytes, eosinophils, basophils and blasts with counts obtained by manual counting. Bias and Allowable error were derived from Bland-Altman analysis, which compared the post-classification absolute counts for neutrophils, monocytes, lymphocytes, eosinophils, basophils and blasts with counts obtained by manual counting;

§ Allowable bias as recommended by the Westgard biodatabase.^12^

### Analysis of misclassification rates

Although only 3.5% of cells were classified as ‘unidentified’ in the pre-classification DWCC, overall 16% required reclassification (Table 3). As the most common cell type, neutrophils were the most frequently misclassified, but overall, only 7.6% of all neutrophils required reclassification. In contrast, close to 30% of monocytes, eosinophils and blasts, as well as > 90% of basophils, were misclassified. Multivariate analysis revealed a significant relationship between the number of misclassified cells and both the WCC and the presence of malignant cells in the blood (Table 4). No other variables analysed, including HIV status, had a significant association with the number of misclassified cells.

**TABLE 3:** Misclassification of cells by CellaVision™ DM96 analyser, Chris Hani Baragwanath Academic Hospital haematology laboratory, Johannesburg, South Africa, 2012–2013 (*N* = 149).

White cell type	Proportion of all misclassifications† (%) (mean[±SD])	Proportion of cell type reclassified‡ (%) (mean[±SD])
All cells	N/A	16.4
Neutrophils	26.7 (±35)	7.6 (±12.3)
Lymphocytes	20.8 (±69.7)	11.9 (±15.3)
Monocytes	11.9 (±15)	28.9 (±31.7)
Eosinophils	3.2 (±8.7)	31.7 (±37.6)
Basophils	2.9 (±7)	93.5 (±23.1)
Blasts	2.4 (±11.8)	33.7 (±38.6)
Other§	32.1 (±73.1)	N/A

NA, Not applicable.

† Calculated as the percentage of all misclassified cells for each type of white cell;

‡ Calculated as the percentage of cells requiring reclassification within each type of white cell;

§ Includes smudge cells, artefacts, nucleated red cells, immature granulocytes.

**TABLE 4:** Associations of variables of interest with misclassification rates of cells by CellaVision™ DM96 analyser, Chris Hani Baragwanath Academic Hospital haematology laboratory, Johannesburg, South Africa, 2012–2013 (*N* = 149).†

Variable of interest	Adjusted *β* co-efficient	Standard error	*p*-value
HIV infection	2.7	2.0	0.17
Evidence of infection	0.72	2.2	0.73
Malignant cells present	8.0	4.0	0.049
History of malignancy	-2.8	3.7	0.45
History of chemotherapy	2.1	3.6	0.56
White cell count	0.06	0.03	0.035

*r*^2^ = 0.17; (*P* < 0.0001)

† Multivariate linear regression analysis was performed to assess the relative impact of variables of interest on misclassification rates. Any data point with a standard residual of greater than 2.5 was excluded from analysis and *p*-values less than 0.05 were considered statistically significant.

## Discussion

In this study, we assessed the performance of the DM96 as compared to a manual DWCC in 149 samples collected from patients with a wide range of infections and haematological pathologies in a large South African state hospital serving a population with high HIV prevalence. Results from similar studies performed in developed countries have varied. Park et al. showed excellent correlation (CC > 0.9) for all cell types except promyelocytes and basophils.^13^ In contrast, although most other studies have also shown good correlations for neutrophils, lymphocytes and blasts, correlations were generally poorer for monocytes (CC 0.67–0.83), eosinophils (CC 0.73–0.88), and basophils (0.05–0.76).^6,7,8,9^ Similarly in our study, there was excellent correlation (CC > 0.95) for both neutrophils and blasts and substantially weaker correlation (CC < 0.75) for eosinophils and basophils. Interestingly, the correlations for both lymphocytes (CC = 0.86) and monocytes (CC = 0.51) were poorer in our study than those reported previously. Nonetheless, we found that agreement was within acceptable limits of bias for both the monocyte and lymphocyte values, and accuracy was thus judged to be adequate for these parameters. The diversity of results between studies may be attributable, in part, to the proportion of pathological samples included in each study. In addition, Park et al. found that correlations improved in samples with low WCCs when the DM96 was preset to analyse a higher number of white cells (300–500), which may have impacted their overall correlation results.^13^ The relatively poorer correlation consistently seen for monocytes, basophils and/or eosinophils is likely a result of the predictably poor precision expected when cells present in small numbers are assessed by a limited cell count. Reassuringly, where discrepancies between the methods were evident in our study, the differences translated into small changes in absolute cell counts, the clinical significance of which was negligible.

Although HIV infection has well documented effects on white cell morphology,^11^ misclassification rates in our study were not associated with HIV status. Misclassification rates were significantly associated with WCC and the presence of malignant cells in the peripheral blood, whereas chemotherapy exposure, a history of prior malignancy and the presence of infection were not. ‘Unidentified cells’ made up 3.5% of cells in our study, but overall, 16% required reclassification. Our misclassification rate was higher than that described previously by Rollins-Raval, Raval and Contis, who assessed the performance of the DM96 over a six-month period at three separate sites.^14^ The number of unidentified cells in that study was ~1% and the number of misclassified cells ranged from 4.6% – 12.7%. The higher misclassification rate in our study is likely a product of the large number of specimens containing malignant cells and selection of a large number of samples with extreme WCCs for the purpose of evaluating the analyser. Thus, samples with abnormal WCCs were disproportionately prevalent in comparison to the routine laboratory workload. The overall accuracy of the DM96 is therefore likely to be better than shown in our study, particularly when the WCC is near normal and malignant cells are not present. That being said, in the current era of sophisticated full blood count analysers able to perform accurate DWCCs in a timely manner, the necessity of performing manual DWCCs is restricted to samples in which the analyser fails to perform an accurate DWCC because of the presence of abnormal white cells. Consequently, the accuracy of results for samples with abnormal cells present is of greatest interest, as these specimens would potentially be eligible for analysis with the DM96 because of the need for a manual DWCC.

The time required to perform a DM96 DWCC has been demonstrated to be less than that required for a manual DWCC,^6,8,9^ which raises hopes for greater laboratory efficiency when a manual DWCC is needed. However, the increased misclassification rate in the presence of abnormal cells shown in our study necessitates reclassification of a greater number of cells, which prolongs the time required to perform a DM96 DWCC in this setting. This could conceivably negate the marginal reduction in the time it takes for experienced morphologists to perform a DWCC using the DM96 as compared to a manual count. Cornet, Perol and Troussard suggested that the DM96 would prove to be time efficient, as the few unidentified cells could be quickly and easily classified and validated, thus reducing the time spent on microscopy by technical staff.^5^ However, we found that the number of unidentified cells comprised only about 20% of the misclassified cells; thus, re-assignment of only the unidentified cells would compromise accuracy to an unacceptable extent. Time efficiency is also undermined in samples with very low WCCs, where accuracy is reportedly increased by pre-setting the analyser to count 300–500 cells. However, this improvement comes at the expense of a longer analysis time.^13^ Moreover, the improvements in cell recognition and flagging technology in automated analysers mean that the vast majority of samples can be reviewed by scanning the peripheral smear without the need for a manual DWCC. Thus, in samples without a substantial number of abnormal cells present, the potential analytical time advantage expected from the DM96 is eliminated by the performance of a ‘smear scan’ in lieu of a manual DWCC. Given the benefits of a smear scan over a manual DWCC in the majority of samples, as well as the poorer time efficiency of the DM96 anticipated in samples with leukopenia or abnormal cells present, the utility of the DM96 is placed in question. This is emphasised by the user-dependent nature of correlation studies shown by Briggs et al., where the accuracy of the DM96 was noticeably poorer when the analysis was performed by less-experienced microscopists.^6^ Clearly, although the DM96 is a brilliant piece of innovative technology, it does not eliminate the need for skilled morphologists.

### Limitations

There are some limitations to our study which should be considered when interpreting the results. A large proportion of our HIV-negative patients had a malignancy, so they cannot be regarded as being representative of the normal population. No normal control group was included, so the accuracy of the DM96 was therefore not assessed in the normal population. Our sample size is relatively small and the number of samples included with extreme WCCs and abnormal cells present most likely skewed our results to some extent.

### Conclusion

This study showed that the performance of the DM96 in an African laboratory serving a population with a high HIV-prevalence was similar to that described in developed countries. However, significant intervention from experienced morphologists remains necessary for the validation of results, particularly when malignant cells are present. Given the substantial cost of this sophisticated instrument, it would be difficult to justify its routine use in a resource-constrained setting.
